# Burnout and Sources of Stress Among Health Care Risk Managers and Patient Safety Personnel During the COVID-19 Pandemic: A Pilot Study

**DOI:** 10.1017/dmp.2021.120

**Published:** 2021-04-19

**Authors:** Alan J. Card

**Affiliations:** Department of Pediatrics, Division of Hospital Medicine, University of California, San Diego School of Medicine, La Jolla, CA, USA

**Keywords:** Covid-19, health services, manpower, occupational health, psychological stress, risk Management

## Abstract

**Objective:**

This study investigates burnout and sources of stress related to the coronavirus disease 2019 (COVID-19) pandemic among a group of health care risk managers/patient safety practitioners.

**Methods:**

An online survey was used, including the Oldenburg Burnout Inventory (OLBI) and 1 open-ended question: Since the start of the COVID-19 pandemic, what work or non-work-related issues have been causing you the most stress?

**Results:**

A total of 31 participants completed the OLBI; 27 answered the open-ended question. Over 70% of participants qualified as burned out. A thematic analysis was used to analyze stressors. Key themes included impacts of social distancing, changing duties and workload, real and potential impacts of the virus (eg, fear of infection for self or others), and financial concerns (personal and organizational). Less common themes included untrustworthy and constantly changing guidance, feeling abused by persons in power, and positive comments about the experience of working during the pandemic.

**Conclusion:**

Burnout and pandemic-related stress may be very common in the health care risk management and patient safety workforce. Additional research is required to more robustly estimate the prevalence of burnout in this population. Meanwhile, the sources of stress identified here may aid health care organizations in taking immediate action to protect this vital workforce.

## Introduction

The coronavirus disease 2019 (COVID-19) pandemic has exacerbated an ongoing epidemic of burnout among health care workers (HCWs). Most literature on pandemic-related stress has rightly focused on clinicians. Studies describing the impact on *non-clinician* HCWs have largely been limited to comparisons between undifferentiated non-clinical staff and frontline clinicians.

This study investigates burnout and sources of COVID-19-related stress among health care risk management and patient safety practitioners – specifically, members of the Southern California Association of Healthcare Risk Management (SCAHRM). This workforce plays an important role in facilitating the delivery of safe and effective care in both routine and disaster response operations. During the current COVID-19 pandemic, for instance, these workers staff incident command centers respond to emerging threats to patient and staff safety, and even engage in contact tracing/exposure notification.

To the author’s knowledge, this is the first study to address the psychological impact of the COVID-19 pandemic in health care risk management and patient safety professionals.

## Methods

An online survey was used, including the Oldenburg Burnout Inventory (OLBI), a validated burnout measure,^1,2^ and 1 open-ended question: Since the start of the COVID-19 pandemic, what work or non-work-related issues have been causing you the most stress?

The OLBI is a 16-item Likert-type scale made up of two 8-item subscales: *exhaustion* and *disengagement*. The instrument is evenly divided between positively and negatively coded items. Negatively coded items relate to the opposite of the subscale constructs (ie, *energy* and *engagement,* as opposed to *exhaustion* and *disengagement*). Survey items are constructed on a 4-point scale, ranging from *strongly agree* to *strongly disagree,* with scoring reversed for negatively coded items. While proprietary, the OLBI is free to use and has been widely applied in studies of the health care workforce. OLBI outcomes were defined by subscale scores, calculated as the mean of the item scores for each subscale (range: 1-4):

*Burnout* – Exhaustion score ≥ 2.25 and disengagement score ≥ 2.1

*Exhausted* – Exhaustion score ≥ 2.25

*Disengaged* – Disengagement score ≥ 2.1

*No burnout* – Exhaustion score < 2.25 and disengagement score < 2.1^1^

Free text responses were assessed using content analysis.^3^

The study was evaluated by the University of California, San Diego Institutional Review Board, and determined to be exempt.

## Results

A total of 31 participants completed the OLBI, and 27 responded to the open-ended question for an overall response rate of 17% of SCAHRM’s 187 members.

### Burnout

Twenty-one (71%) participants had *burnout.* Twenty-one (71%) were *disengaged*, all of whom also had burnout. Twenty-four (77%) were *exhausted* (emotionally), 3 (10%) of whom did not qualify as burned out. Six (19%) had *no burnout*.

### Sources of Stress

*Impacts of social distancing* (especially the lack of personal interaction) tied for the most common theme, appearing in 48% of responses. Subthemes included work-related and non-work-related concerns, as well as the interface between the 2 (eg, effects of working from home). Outside of work, the inability to relieve stress in accustomed ways (“…not getting to go out and do the things I enjoy – shopping, church, the gym, travel, seeing family and friends”) and concerns about the social isolation of family members were prominent themes. Difficulties managing family obligations while working from home were also an important issue – especially for the parents of school-aged children (eg, “I think the greatest challenge has been learning to work from home with my kids home! March and April were VERY difficult.”).

Regarding the effects of social distancing on work, participants described impaired communication when working from home, as well as degraded work relationships with peers and managers (eg, “When we do not see each other and are not able to interact, relationships breakdown [sic].”). A few participants also described infrastructure challenges, such as non-ergonomic workspaces or difficulties with information technology.

The broad category of *changing duties and workload* tied with *social distancing* for the most common theme. Reported by 48% of participants, this theme covered a diverse array of impacts. Among the most common were issues related to *emotional labor*. This included dealing with increased stress and burnout among staff/managers; dealing with difficult interactions with patients and families (eg, “constant patient family complaints/concerns regarding communication”); and calling people to provide exposure notification:

*Work now includes calling patients or providers who have been exposed to covid while working or present in the facility. Additionally, we are calling covid lab results to our patients who have tested in our emergency department who do not have access to their labs online. Even though they have had treatment and after-care education by excellent teams, they still want to vent and are scared of their results. Additionally, it is stressful to hear their stories about how they have not socially distanced which means more patients will be coming our way.*

Many respondents also described the need to cover for staffing cuts and absenteeism.

Some participants reported new duties (eg, exposure notification, as noted above) and changes to work hours (“We are pretty much on-call 7 days a week and frequently work 12-hour days when we have a list of patients to call”). Less common subthemes included an increased number of adverse events, reduced organizational capacity for handling ordinary safety and compliance work, and the need to develop policies for ethically challenging situations.

*Real and potential impacts of the virus*, itself, were another important theme, accounting for 19% of responses. The most common subtheme related to fear of family members contracting COVID-19 (eg, “Constantly trying to choke backs our own personal fears of contracting COVID at work and bringing it home to my family and my elderly mom who lives with me”). Other subthemes included fear of becoming ill, the emotional toll of patient deaths, and the struggle to keep patients and staff safe from the virus. Failure to follow guidelines on the part of both the general population and staff members was an important part of this last challenge (eg, “One major stress is hearing that some HCWs have continued to work while symptomatic by basically lying at our checkpoints, thereby exposing others unnecessarily. This unethical behavior is by far the biggest stress I have.”)

Financial concerns (both personal and organizational) were an important theme identified by 26% of participants (eg, “impact of unemployment of family members on me and fear if I lose my job” and “Severe cuts in leadership due to financial shortfalls are my most stressful work-related issue.”). One respondent had already been laid off. Another feared losing her/his job, and many described covering for staffing cuts.

The 3 remaining themes were less common. One was *untrustworthy and constantly changing guidance*, which was identified by 11% of respondents (eg, “Dealing with the constant changes in directives from the CDC [The Centers for Disease Control and Prevention] and Government agencies when it comes to guidance that can literally change overnight,” and “The lack of consistency of rules for business and schools throughout the state. Many high risk activities seem to be permitted while others are not.”). Another theme, reported by 4% of participants, was *feeling abused by persons in power*. Finally, 11% of participants responded with positive comments about their work during the pandemic, especially regarding management support.

## Discussion

Burnout was extremely common, affecting > 70% of participants. The sample size (n = 31) was fairly small. Additional research is needed to more robustly estimate the burnout rate among health care risk management and patient safety personnel. However, the findings of this pilot study are sufficient to suggest that burnout is an important issue in this population and that further research is warranted.

Recent attention to burnout among clinicians^4,5^ reflects an overdue acknowledgment that the goals of health care safety apply to *everyone* in the health care ecosystem. This article expands that logic to non-clinical HCWs, focusing on members of the health care quality and safety workforce, a population that has been mostly (but not entirely) neglected.^6,7^

Among clinicians^4^ and other workers,^8^ burnout is associated with impaired work performance. If this holds true among members of the health care risk management and patient safety workforce, burnout among these professionals represents a threat to the delivery of safe and effective care, both during the pandemic and in the recovery phase, when posttraumatic stress disorder may impose a continuing burden. Turnover related to occupational stress may also lead to poorer outcomes, given the dearth of experienced risk and safety professionals prepared to step into these roles.^6^

The pandemic-related stressors identified by the participants of this study present important targets for both prevention and mitigation. They broadly align with the “psychological triggers” identified by Meredith et al.^9^ as important drivers of mental health during large-scale disasters. These include *restricted movement, limited resources, trauma exposure, limited information,* and *perceived personal or family risk.*

In designing interventions to address burnout, it is important to differentiate between sources of avoidable suffering (issues with work design, which should be addressed through systems improvement) and unavoidable suffering (which should be addressed by promoting individual resilience).^5,10^ Expecting workers to shoulder the burdens of unsafe or unnecessarily stressful working conditions through “grit” or resilience is “…an unethical abdication of duty on the part of health care managers.”^5^ Many sources of pandemic-related stress, however, are beyond the organization’s scope of control. In these cases, interventions aimed at individual resilience may be warranted.

While additional research is clearly warranted, the findings presented here have led the professional association that hosted the survey to begin designing a peer support program for its members. Health care organizations and other stakeholders should follow suit by taking immediate action to reduce burnout among health care risk management and patient safety personnel. In the longer term, researchers should investigate the causes and consequences of burnout in this population (and among non-clinical HCWs, more generally).
